# Reviewed article: Campos-Rosa J, Puchivailo RAC, Schäfer AA, Meller FO, Miranda VIA. Ferrous Sulfate and Folic Acid Supplementation in Pregnant People in Primary Health Care: a Cross-Sectional Study, Criciúma, 2022. Epidemiol Serv Saude. 2026:35;e20240377

**DOI:** 10.1590/S2237-96222026v35e20240377.a

**Published:** 2025-11-28

**Authors:** Cátia Regina Ficagna

**Affiliations:** 1 Universidade Federal do Rio Grande do Sul, Porto Alegre, RS, Brazil Universidade Federal do Rio Grande do Sul Porto Alegre RS Brazil

## Peer review A

## Questionnaire

Does the manuscript contain new and significant information to justify publication? Yes

Does the Abstract (Summary) clearly and accurately describe the content of the article? Yes

Is the problem significant and concisely stated? Yes

Are the methods described comprehensively? Yes

Are the interpretations and conclusions justified by the results? Yes

Is adequate reference made to other work in the field? Yes

## Manuscript Structure

Length of article is: Adequate

Number of tables is: Adequate

Number of figures is: Adequate

Please state any conflict(s) of interest that you have in relation to the review of this paper. None

Interest: Good

Quality: Average

Originality: Good

Overall: Good

Recommendation: Minor Revision

## Comments

Introdução: ok

Métodos: Na seção coleta de dados tem um erro de digitação, quando escrevem o nome do aplicativo RedCap - Research Electronic Data Capture.

Talvez seria interessante reescrever essa frase: “Os desfechos “suplementação de ferro” e “suplementação de ácido fólico” foram retirados de campo específico da carteira de pré-natal das gestantes”. Descrever como eram feitos esses registros...

Discussão: O segundo parágrafo está um pouco confuso. Sugiro reescrever. Podem começar, se preferirem, com a parte que fala sobre a tendência para o maior uso de suplementação de ferro nas fases iniciais....

No terceiro parágrafo fala novamente de Pelotas (duas vezes) e sempre é escrito que é no estado do Rio Grande do Sul. Acho que daria pra organizar de maneira que isso não fique repetitivo. Ainda, do jeito como está escrito, dá a impressão de que só Pelotas e Rio Grande realizaram esudos sobre a temática!

